# Relationship between myocardial scar and coronary artery plaque in diabetes patients: from preliminary results of assessment with cardiac computed tomography angiography and magnetic resonance imaging in patients with type 2 diabetes for detection of unrecognized myocardial scar in subclinical coronary atherosclerosis (ACCREDIT) study

**DOI:** 10.1186/1532-429X-15-S1-M4

**Published:** 2013-01-30

**Authors:** Joon-Won Kang, Sang Il Choi, Sung Min Ko, Yeon Hyeon Choe, Byoung Wook Choi, Whal Lee, Tae-Hwan Lim

**Affiliations:** 1 Department of Radiology, Asan Medical Center, Seoul, Republic of Korea; 2 Seoul National University Bundang Hospital, Seongnam, Republic of Korea; 3 Konkuk University Hospital, Seoul, Republic of Korea; 4 Samsung Medical Center, Seoul, Republic of Korea; 5 Severance Hospital, Seoul, Republic of Korea; 6 Seoul National University Hospital, Seoul, Republic of Korea

## Background

Myocardial scar is known as the risk factor for the poor prognosis in the asymptomatic diabetes patients. However, the relationship between the coronary artery disease and myocardial scar is not known. Therefore, the purpose of this study is to evaluate the correlation between myocardial scar on delayed enhancement MRI (DE-MRI) and coronary atherosclerosis on CT coronary angiography (CTCA) in asymptomatic patients with type 2 diabetes.

## Methods

In this prospective, multicenter, and open-label study, 347 patients with type 2 DM were included who had 2 or more risk factors of coronary artery disease (CAD). DE-MRI and CTCA were performed on 322 patients. The prevalence of OMS and total number of involved myocardial segments on DE-MRI were evaluated. And then, the association of presence of OMS with clinical parameters (age, gender, body mass index, duration of diabetes, dyslipidemia, family history, smoking history, and HbA1c level) and coronary calcium score. Association of OMS and the degree of stenosis and plaque characteristics of coronary artery on CTCA per patient was also evaluated.

## Results

OMS was detected by CMR in 23(7.1%) of the 322 patients and the number of myocardial scar is 49 (median 2, range 1-4 scars per patient with OMS). The prevalence of CAD was 74.5% (240 of 322), and the more than 50% stenosis was found in 24.9% of the subjects (80 of 322). Using logistic regression, only coronary calcium score more than 100 was the predictor of presence of OMS (odds ratio 7.484, 2.459-22.776 in 95% confidence interval). The prevalence of OMS was significantly higher in patients with more than 50% stenosis (p<0.001 using Chi-square test) and in patients with mixed and non-calcified plaques.

## Conclusions

The prevalence of OMS on DE-MRI and significant coronary disease on CTCA are not uncommon even in asymptomatic patients with type 2 diabetes. Coronary calcium score more than 100 is the only predictor of OMS.

## Funding

This study was supported by Guerbet Group;

